# Antioxidant Response of *Lepidium sativum* L. to Metallic Trace Element Stress Under Controlled Mycorrhization Conditions

**DOI:** 10.1002/fsn3.72251

**Published:** 2026-08-10

**Authors:** Hanae El Yeznasni, Wissame Chafai, Elmostafa Gagou, Mashail N. Alzain, Omar Noman, Fahd A. Nasr, Mohamed Addi, Ahmed Khalid

**Affiliations:** ^1^ Laboratory for the Improvement of Agricultural Production Biotechnology and Environment University Mohammed the First Oujda Morocco; ^2^ Laboratory of Bio Resources, Biotechnology, Ethno Pharmacology and Health, Faculty of Sciences University Mohamed Premier Oujda Morocco; ^3^ Department of Biology, College of Science Princess Nourah Bint Abdulrahman University Riyadh Saudi Arabia; ^4^ Department of Pharmacognosy, College of Pharmacy King Saud University Riyadh Saudi Arabia; ^5^ Biology Department, College of Science Imam Mohammad Ibn Saud Islamic University (IMSIU) Riyadh Saudi Arabia

**Keywords:** antioxidant activity, arbuscular mycorrhizal fungi (AMF), glomalin‐related soil proteins, heavy metal stress, lead (Pb), *Lepidium sativum*, phytoremediation, plant tolerance

## Abstract

Abandoned mining sites are major sources of metallic trace element contamination, which negatively affects plant growth, physiological functions, and metabolic activities. In addition, stress caused by metallic trace elements can alter the biosynthesis and accumulation of secondary metabolites. Among the various soil remediation strategies, phytoremediation assisted by arbuscular mycorrhizal fungi (AMF) has emerged as a promising approach. This research examined how effective native AMF are in improving the tolerance of *Lepidium sativum L*. to lead (Pb) stress. The plants were first cultivated for 2 months in uncontaminated soil before being exposed to 50 mg L^−1^ Pb in a controlled environment. A comparative analysis was carried out to assess the impact of mycorrhizal inoculation on plants subjected to Pb exposure versus those that were not. The effects of mycorrhization on growth, physiological performance, and biochemical responses were evaluated. The results demonstrated that mycorrhization positively influenced plant responses to lead stress, as evidenced by improved growth and physiological performance. Compared with non‐mycorrhizal plants, mycorrhizal plants exhibited increases of approximately 36% and 17% in chlorophyll a and chlorophyll b contents, respectively, as well as an approximately twofold increase in total soluble sugars. Furthermore, mycorrhization promoted proline accumulation and significantly enhanced catalase activity, with mycorrhizal plants showing an approximately 55% increase compared with non‐mycorrhizal plants, indicating a greater capacity to alleviate oxidative stress. Mycorrhization also increased the production of glomalin‐related soil proteins in the rhizosphere, further contributing to plant tolerance under lead stress. These findings highlight the potential of indigenous AMF as an eco‐friendly and sustainable strategy for enhancing plant tolerance and promoting phytoremediation in lead‐contaminated soils.

## Graphical abstract

*Lepidium sativum* plants were inoculated with arbuscular mycorrhizal fungi (AMF) and exposed to Pb stress (50 mg L^−1^). Catalase activity, soluble sugars, glomalin, proline, and chlorophyll a and b were analyzed. Overall, mycorrhizal plants showed the most favorable biochemical responses under Pb stress, highlighting the beneficial role of AMF in Pb tolerance.

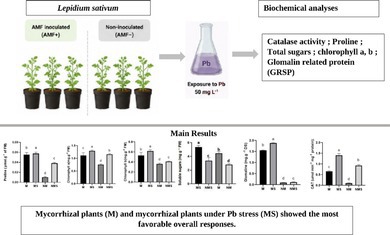

## 1 Introduction

The contamination of ecosystems by trace elements (TEs) has been recognized as a major threat to environmental and human health due to their toxicity and persistence in the environment (Sharma et al. 2023). Trace elements originate from both natural processes and various anthropogenic activities. Because they are nonbiodegradable, they can remain in soils for decades after contamination, increasing the risk of transfer through food chains and causing adverse effects at different biological levels (Yan 2020). Some trace elements, such as zinc (Zn), iron (Fe), and manganese (Mn), are essential micronutrients involved in plant metabolism; however, excessive accumulation may result in phytotoxicity (Chaney 1993; Fernando and Lynch 2015). Zinc toxicity is generally observed when concentrations exceed 500 mg kg^−1^ dry weight (DW) in plant tissues, while iron toxicity may occur at foliar concentrations above 500 mg kg^−1^ dry DW. Manganese toxicity symptoms are commonly reported at foliar concentrations ranging from 400 to 1000 mg kg^−1^ DW, depending on plant species and environmental conditions (Fernando and Lynch 2015; Labudda and Azam 2019). In contrast, lead (Pb), cadmium (Cd), and arsenic (As) have no known physiological function in plants and can exert toxic effects even at relatively low concentrations (Siqueira‐Silva et al. 2018; Labudda and Azam 2019). In Morocco, metallurgy has been a major source of trace element (TE) contamination since the beginning of industrialization. Numerous metalliferous mining sites were intensively exploited and subsequently abandoned, leaving behind significant environmental pollution (Midhat et al. 2019). The ecological risks associated with these sites persist long after mining activities cease because trace elements remain in the soil and continue to accumulate in local vegetation (Priyadarshini et al. 2021). Among these contaminants, lead (Pb) is considered one of the most hazardous due to its persistence and toxicity. To address TE‐contaminated soils, several remediation technologies have been developed worldwide. Among them, the use of AMF has emerged as a cost‐effective and environmentally friendly strategy for enhancing phytoremediation efficiency (Yang et al. 2022). AMF are able to improve plant tolerance to metal stress through a range of physiological and biochemical mechanisms (Andrade et al. 2009). Metal exposure can also select for tolerant fungal communities, resulting in the predominance of strains adapted to contaminated environments (Akhtar et al. 2024; Riaz et al. 2021). In addition to mitigating stress, AMF contribute to plant nutrition by enhancing nutrient acquisition, particularly through improved phosphorus availability and nitrogen cycling processes (Li et al. 2023).

The use of AMF as biofertilizers has gained increasing attention because of their ability to improve soil quality, promote root development, and enhance plant tolerance to environmental stress. In phytoremediation programs, the inoculation of nonfood, medicinal, or indicator plant species represents an attractive strategy, as these plants often exhibit high tolerance to trace elements (TEs) while minimizing risks associated with food‐chain contamination (Pirsarandi et al. 2022). AMF employ a range of metabolic and non‐metabolic mechanisms to cope with TE stress, making mycorrhizoremediation a promising and environmentally sustainable bioremediation technology (Hachani et al. 2020).

In this context, *Lepidium sativum* L. ( *L. sativum* ) was selected as a model species. Native to Ethiopia, Eritrea, and Southwest Asia, it has been cultivated since ancient times and is now widely naturalized worldwide (Wadhwa et al. 2012; Sharma 2020). This fast‐growing annual herb of the Brassicaceae family is valued for its nutritional and medicinal properties (Mossa et al. 1994) and is frequently used in phytotoxicity studies because its germination and root growth are highly sensitive to metal contamination (Leszczyńska and Smolińska 2025; Mizerna et al. 2021).

The present study aimed to evaluate the effects of indigenous AMF inoculum derived from mining soils on the growth, physiological performance, and stress responses of *Lepidium sativum* L. exposed to lead (Pb) contamination under controlled conditions. A comparative experiment was conducted using mycorrhizal and non‐mycorrhizal plants grown under both Pb‐stressed and control conditions. Mycorrhizal parameters, growth parameters, proline and total soluble sugar contents, chlorophyll a and chlorophyll b concentrations, antioxidant enzyme activity, and glomalin‐related soil protein production were assessed.

## 2 Material and Methods

### 2.1 Inoculum Preparation From Mining Site‐Derived Fungal Material

The fungal inoculum used in the present study was obtained from the roots of an onion crop ( *Allium cepa* L.) cultivated in soil contaminated with mine waste from the Oued El Heimer mining site for 6 months. After harvesting the crop, the onion roots were air‐dried at ambient laboratory conditions (approximately 25°C ± 2°C) for 5–7 days until a constant weight was reached. The drying was carried out in a well‐ventilated area, protected from direct sunlight to preserve the viability of the inoculum. This trapping method was used to propagate indigenous AMF originating from the contaminated site. After microscopic identification of mycorrhizal colonization, root fragments ranging from 1 to 2 mm were observed with a mycorrhizal frequency and intensity of 75% and 70%, respectively.

### 2.2 Evaluation of Mycorrhizal Colonization Rate

The assessment of mycorrhization parameters was carried out by observing thirty root fragments of approximately 1 cm in length, randomly selected in order to quantify mycorrhizae. These fragments were mounted in parallel groups of ten in a drop of glycerinated water between a slide and a coverslip. Subsequently, a direct count was performed on all mounted fragments. Each fragment was carefully examined along its entire length under ×100 and ×400 magnifications.

The frequency and intensity of mycorrhizal colonization in the inoculum roots were determined according to the method of Trouvelot et al. (1986). The frequency of mycorrhization (F%) was calculated using the following equation:

$$F\%=\left[\left(N-N_{0}\right)/N\right]\times 100$$

where: *N* = total number of root fragments observed; *N*_0_ = number of root fragments with no mycorrhizal structures.

The intensity of root cortex colonization (M%) was calculated using the following equation:

$$M\%=\left[\left(95\times \text{n5}\right)+\left(70\times \text{n4}\right)+\left(30\times \text{n3}\right)+\left(5\times \text{n2}\right)+n\right]/N$$

where: *N* = total number of root fragments observed; n1–n5 represents the number of fragments classified into a scale from 0 to 5, depending on the proportion of the fragment's cortex colonized. For example, 100 > n5 > 90; 90 > n4 > 50; 50 > n3 > 10; 10 > n2 > 1; and 1 > n1 > 0.

### 2.3 Plant Material

Seeds of *L. sativum* were surface‐sterilized by soaking in a solution containing 10% sodium hypochlorite for 5 min, followed by repeated rinsing in sterile distilled water. The seeds were then sown in pots containing autoclaved peat. For mycorrhizal treatments, 1 g of prepared dried root inoculum was mixed into the substrate during sowing, while control plants were grown in the absence of any inoculum. After a growth period of 2 months, mycorrhizal association formation in some of the inoculated plants was confirmed using a staining technique with trypan blue, as per Phillips and Hayman (1970). After confirmation of mycorrhizal colonization, a lead stress of 50 mg L^−1^ Pb (in Pb nitrate solution) was applied to half of the mycorrhizal and non‐mycorrhizal plants, while the remaining plants were not subjected to any lead stress. Therefore, in this experiment, four different lead stress levels were applied:

– Control (NM): Non‐mycorrhizal plants not subjected to lead stress
– Mycorrhizal (M): Plants subjected to mycorrhizal association formation, not subjected to lead stress
– Control + Pb (NMS): Non‐mycorrhizal plants subjected to lead stress
– Mycorrhizal + Pb (MS): Plants subjected to mycorrhizal association formation and lead stress

### 2.4 Physiological and Biochemical Analyses

#### 2.4.1 Chlorophyll Measurement

Quantification of photosynthetic pigments was quantified according to the protocol of Wellburn and Lichtenthaler (1984). Fresh leaf samples (0.1–0.2 g) were removed from the plant, fragmented, and added to a test tube containing 5 mL of absolute methanol (96%–100%). The tubes were incubated in the dark for a period of 2 h. Absorbance was measured using a FlexA‐200/FlexA‐200HT microplate reader (Allsheng Instruments, China) at 470 nm, 653 nm, and 666 nm. The chlorophyll a and chlorophyll b contents were calculated using the following equations:

$$\text{Chlorophyll}\;a\;\left(C_{a}\right)=15.65\;A_{666}-7.340\;A_{653}$$

$$\text{Chlorophyll}\;b\;\left(C_{b}\right)=27.05\;A_{653}-11.21\;A_{666}$$

#### 2.4.2 Proline Measurement

Proline concentration was determined according to the method described by Bates et al. (1973) with slight modifications. Fresh leaves (0.2 g) were homogenized with 40% v/v methanol. To this homogenate, 1 mL of ninhydrin reagent, which is prepared by dissolving 1% w/v ninhydrin in a solvent system containing 60% v/v glacial acetic acid and 20% v/v ethanol, was added. This mixture was then heated at 92°C for 20 min. Proline concentration was determined after cooling the mixture by measuring the absorbance at 530 nm using a spectrophotometer.

#### 2.4.3 Total Soluble Sugar Quantification

The total soluble sugar concentration was determined using a modified anthrone method, as described by Dubois et al. (1951). Fresh leaves (0.1 g) were placed in 2 mL of anhydrous ethanol and incubated in the dark for 24 h. After this, the extract was directly diluted tenfold with anhydrous ethanol without evaporation prior to analysis. Subsequently, 4 mL of a freshly prepared solution of anthrone 2% (w/v) in sulfuric acid was mixed with the solution, vortexed, and incubated in a water bath at 92°C for 8 min and then left to cool in the dark for 30 min.

#### 2.4.4 Catalase Activity Assay

Catalase activity was measured by the spectrophotometric determination of the rate of hydrogen peroxide degradation according to the method of Aebi (1984). The enzyme extract was obtained by homogenizing fresh plant material in 50 mM phosphate buffer (pH 7.0) using a mortar and pestle. The homogenate was then centrifuged at 5000 rpm for 20 min at 4°C using a Z 327 K refrigerated centrifuge (Hermle Labortechnik, Germany), and the resulting supernatant was used as the enzyme extract. The reaction mixture contained 50 mM phosphate buffer (pH 7.0) and 10 mM H_2_O_2_, to which the enzyme extract was added to initiate the reaction. The decrease in absorbance at 240 nm, reflecting H_2_O_2_ degradation, was monitored for 1 min at room temperature using a FlexA‐200/FlexA‐200HT microplate reader (Allsheng, China). Catalase activity was calculated using the molar extinction coefficient of H_2_O_2_ (ε = 39.4 mM^−1^ cm^−1^) and expressed as U mg^−1^ protein. Protein content in the enzyme extract was determined according to the Bradford method using bovine serum albumin (BSA) as a standard.

#### 2.4.5 Glomalin Extraction and Quantification

Glomalin‐related soil proteins (GRSP) were extracted using the method proposed by Singh et al. (2013). Soil samples were collected from each pot and air‐dried at 35°C until a constant dry weight was achieved. One gram of air‐dried soil from each pot was mixed with 8 mL of 20 mM sodium citrate buffer at pH 7.0 and autoclaved at 121°C for 60 min. After autoclaving, the mixture was centrifuged at 5000 rpm for 10 min to obtain the supernatant, which is considered the readily extractable glomalin‐related soil protein. Protein concentration in the supernatant was estimated using the Bradford protein assay with bovine serum albumin as the standard protein. The absorbance was measured at 595 nm and expressed as milligrams of glomalin per gram of dry soil.

### 2.5 Statistical Analysis

All data were subjected to statistical analysis using statistical software (SPSS, version 21.0, SPSS Inc., Chicago, USA). To determine the effect of two fixed factors, namely mycorrhization (two levels: with and without) and lead stress (two levels: with and without), two‐way analysis of variance (two‐way ANOVA) was carried out on all data sets. When differences were found to be significant (*p* < 0.05) using ANOVA, differences in means among treatments (NM M, NMS, MS) were determined using a Student–Newman–Keuls (SNK) test. Data are expressed as means with their corresponding standard errors, and differences among treatments are represented by different letters (e.g., a, b, c).

## 3 Results

### 3.1 Assessment of Mycorrhizal Colonization

Microscopic analysis of stained root samples of *L. sativum* confirmed the formation of a functional symbiosis with AMF. The presence of characteristic AMF structures, i.e., intraradical hyphae, vesicles, and arbuscules, was observed within the cortical cells of inoculated *L. sativum* plants. In contrast, no structures of AMF were observed within the root systems of non‐inoculated control plants (Figure 1).

**FIGURE 1:**
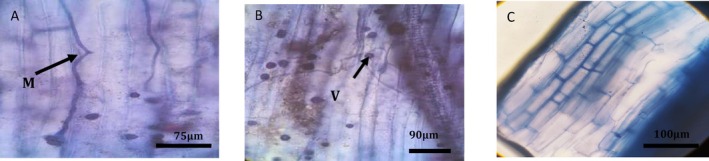
Microscopic observations of root colonization of *Lepidium sativum* plants under 50 mg/L of lead; (A, B) Mycorrhizal plants (C) Control plant; (V) Vesicles; (M) Mycelial network.

Quantitative measurements of mycorrhizal colonization in inoculated *L. sativum* plants are presented in Table 1. Both colonization frequency and colonization intensity exceeded 70% and 80%, respectively, indicating the successful establishment of symbiosis with AMF. The number of vesicles in mycorrhizal plants reached 120 per ten fragments, whereas plants subjected to lead stress showed 115 vesicles per ten fragments. Similarly, a high number of AMF spores was recorded in the substrate of the (M + MS) treatment, with values of 90 and 85, respectively, demonstrating the successful propagation and reproductive capacity of the AMF symbionts.

**TABLE 1:** Mycorrhizal characteristics of inoculated plants.

Characteristics	M	MS
Spores number (per ten fragments)	90^a^	85^a^
Vesicles number (per ten fragments)	120^a^	115^b^
Frequency of mycorrhization	73%^a^	75%^a^
Intensity of mycorrhization	85%^b^	88%^a^
Arbuscules number (per ten fragments)	2^b^	5^a^

*Note:* Different letters within the same row indicate significant differences at *p* < 0.05.

Abbreviations: M, Mycorhizal plants; MS, Mycorhizal and stress.

### 3.2 Arbuscular Mycorrhizal Fungal Community Composition

Figure 2 shows a dominance of the genus *Glomus*, particularly the species *Glomus macrocarpum*, which ranks first with a frequency of 14%, followed by *Glomus citriculata* (10%). These species represent the most frequent taxa in the studied soil. The genera *Acaulospora* and *Scutellospora* are present at lower frequencies (below 8%), indicating moderate diversity but dominated by a few species well adapted to the site conditions. This distribution suggests that the soil favors *Glomus* spores, which are often associated with disturbed environments or mining soils under rehabilitation. The taxonomic composition of the arbuscular mycorrhizal fungal population in the Oued El Heimer mining soil has been established by El Yeznasni et al. (2025).

**FIGURE 2:**
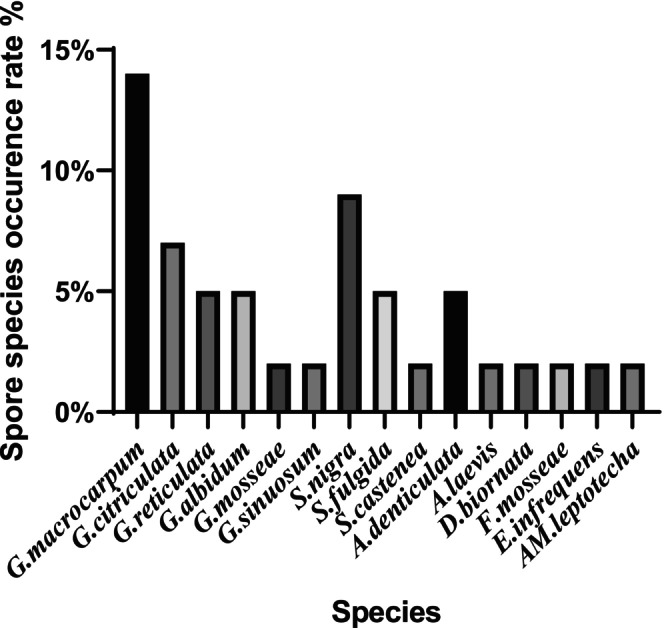
Occurrence of spore species in a mining soil.

### 3.3 Effect of Mycorrhization on Plant Growth Parameters Under Lead Stress

In addition, the evaluation of morphological traits revealed significant differences between inoculated and non‐inoculated plants (Table 2). Under normal growth conditions, plants associated with arbuscular mycorrhizal fungi exhibited significantly greater growth performance than the non‐inoculated control plants. Notably, the height of mycorrhizal plants was more than twice that observed in non‐mycorrhizal plants. Moreover, mycorrhizal inoculation significantly improved both shoot and root fresh weight, resulting in higher fresh weights compared with the control plants.

**TABLE 2:** Stem height (cm); Fresh weight (g/plant) of shoot and root systems of *L. sativum* exposed to lead stress in presence and absence of arbuscular mycorrhizal fungi (AMF) of Oued El Heimer.

Treatment	Plant height (cm)	Fresh weight (g/plant)	Dry weight (g/plant)
Mycorrhizal (M)	7.4ª	1.3ª	0.95^a^
Control (NM)	3.7^c^	0.48ᵇ	0.05^c^
Mycorrhizal and stress (MS)	5.7^b^	1.1^a^	0.43^b^
Control and stress (NMS)	2.1^d^	0.45^b^	0.17^c^

*Note:* Different letters within the same column indicate significant differences at *p* < 0.05.

### 3.4 Proline Accumulation

The proline content in leaf tissues varied noticeably according to the different treatments applied (Figure 3). Mycorrhizal plants showed a substantial increase in proline accumulation compared with non‐mycorrhizal control plants, reaching values of 0.055 and 0.010 μmol/g FM, respectively. This indicates that mycorrhization enhanced proline content by nearly a fivefold increase in proline content as a result of mycorrhizal inoculation. Furthermore, a proportional increase in proline content was also seen in response to stress levels, and the highest levels were found in mycorrhizal plants subjected to lead stress. A two‐way ANOVA was performed to evaluate the effects of mycorrhization and stress, and their interaction, on proline content. The results showed a significant main effect of mycorrhization (*p* < 0.001), with mycorrhized plants showing different proline levels compared to non‐mycorrhized plants. A significant main effect of stress was also observed (*p* < 0.001), indicating that proline content varied significantly between stressed and non‐stressed plants. In addition, there was a significant interaction between mycorrhization and stress (*p* < 0.001), suggesting that the effect of stress on proline accumulation depends on whether the plants are mycorrhized or not.

**FIGURE 3:**
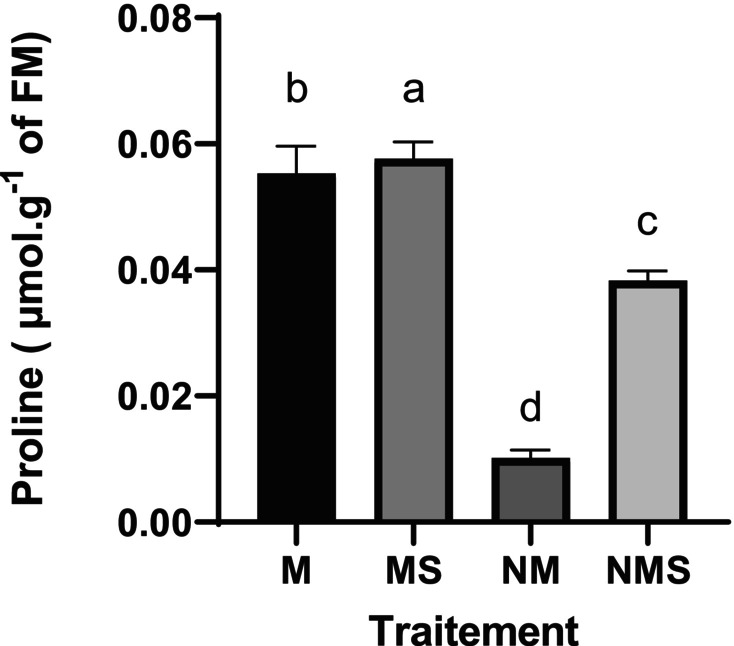
*Lepidium sativum* plants proline content. (NM) Control plants; (M) Mycorrhizal plants; (MS) Mycorrhizal and stressed plants; and (NMS) Control and stressed plants. Different letters indicate statistically significant differences (*p* < 0.05).

### 3.5 Photosynthetic Pigment Content

Lead stress affected the photosynthetic pigments, as shown by the reduction in chlorophyll a and chlorophyll b contents in non‐mycorrhizal plants under Pb stress (Figure 4). Chlorophyll a decreased from 1.16 to 0.75 mg/g FM, while chlorophyll b decreased from 0.41 to 0.36 mg/g FM. In contrast, mycorrhizal plants maintained higher chlorophyll levels under Pb stress, with chlorophyll a and b values reaching 1.18 and 0.57 mg/g FM, respectively, compared with 1.11 and 0.53 mg/g FM in non‐stressed mycorrhizal plants. A two‐way ANOVA showed a significant effect of mycorrhization (*p* < 0.001) and stress (*p* = 0.009) on chlorophyll content, while the interaction between both factors was not significant (*p* > 0.05). The highest chlorophyll a and b contents were recorded in stressed mycorrhizal plants (MS), whereas the lowest values were observed in non‐mycorrhizal non‐stressed plants (NM).

**FIGURE 4:**
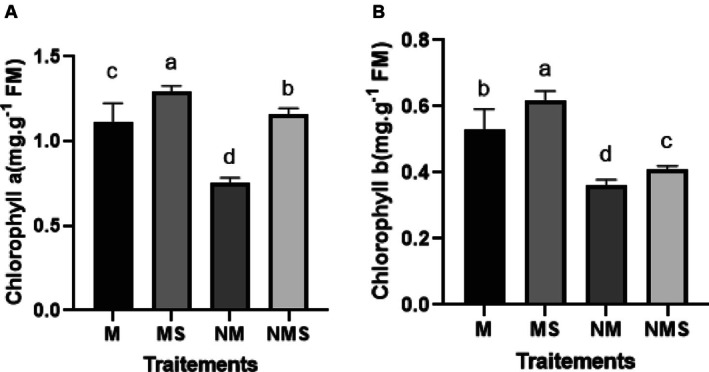
Impact of Lead (P) and Mycorrhization on chlorophyll (A) and chlorophyll (B) content. M, Mycorrhizal plants; MS, Mycorrhizal and Pb‐stressed plants; NM, Non‐mycorrhizal control plants; NMS, Non‐mycorrhizal and Pb‐stressed. Different letters indicate statistically significant differences (*p* < 0.05).

### 3.6 Total Soluble Sugar Content

In addition, analysis of total soluble sugars revealed that distinct effects of treatment were obtained (Figure 5). Mycorrhizal plants exhibited significantly higher total soluble sugar contents than non‐mycorrhizal plants under both control and Pb‐stressed conditions. A two‐way ANOVA was conducted to assess the effects of mycorrhization and stress on total sugar content in *L. sativum* plants. The analysis revealed a significant effect of mycorrhization on sugar content (*p* = 0.002). In addition, the overall model was significant (*p* = 0.009), accounting for 75.0% of the total variation observed (*R* ^2^ = 0.750).

**FIGURE 5:**
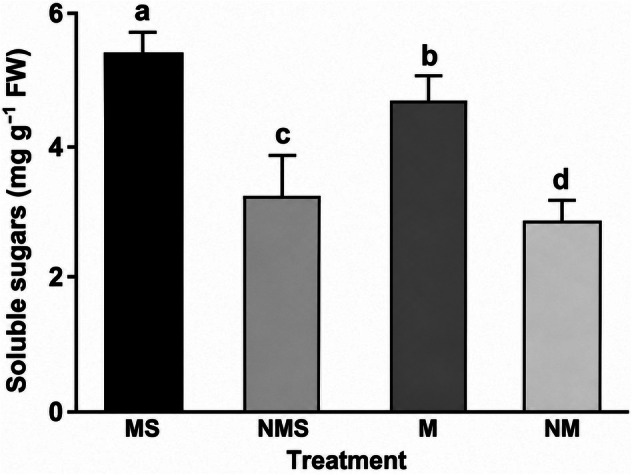
Impact of lead on soluble sugars content. M, Mycorrhizal plants; MS, Mycorrhizal and stressed plants; NM, Control plants; NMS, Control and stressed plants. Different letters indicate statistically significant differences (*p* < 0.05).

### 3.7 Glomalin Content

Significantly higher levels of easily extractable glomalin‐related soil proteins (EE‐GRSP) were found in the rhizosphere soil of mycorrhizal plants compared to non‐mycorrhizal controls, irrespective of lead stress (Figure 6). Soils from pots with non‐mycorrhizal plants showed very low levels of glomalin. The two‐way ANOVA analysis revealed a significant effect of mycorrhization on glomalin content (*p* = 0.001). In addition, the overall model was significant (*p* = 0.007), accounting for 76.0% of the total variation observed (*R* ^2^ = 0.763).

**FIGURE 6:**
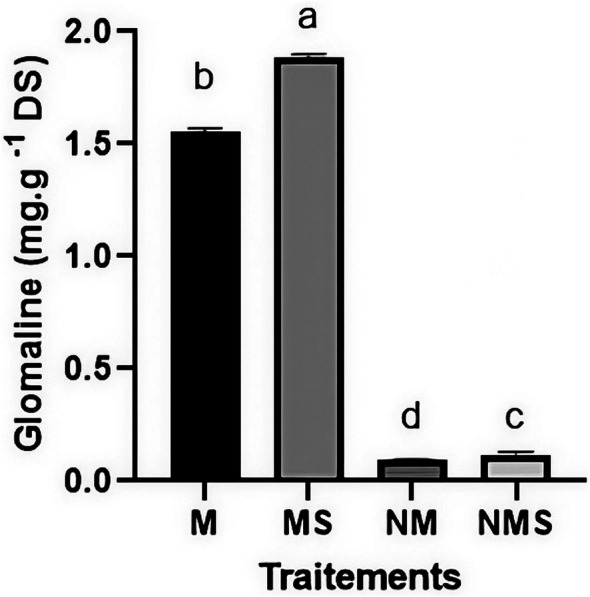
Concentration of EE‐GRSP in soil under different treatments. DS, Dry soil; M, mycorrhizal plants; MS, mycorrhizal and stressed plants; NM, control plants; NMS, control and stressed plants. Different letters indicate statistically significant differences (*p* < 0.05).

### 3.8 Catalase Activity

The catalase (CAT) activity, which is an enzymatic marker for antioxidant defense, exhibited significant variation among the treatments (Figure 7). A sharp increase in the activity of the antioxidant enzyme, ranging from 1.4 to 0.92 (μmol min^−1^ mg^−1^ protein), was noted for mycorrhizal plants under lead stress, followed by non‐mycorrhizal plants under metal stress. However, it is important to note that mycorrhizal plants showed high catalase activity compared to non‐mycorrhizal plants under similar stress conditions. The two‐way ANOVA analysis revealed a significant effect of mycorrhization, stress, and the interaction between mycorrhization and lead stress on CAT activity (*p* < 0.001). In addition, the overall model was significant (*p* < 0.001), accounting for 98% of the total variation observed (*R* ^2^ = 0.989).

**FIGURE 7:**
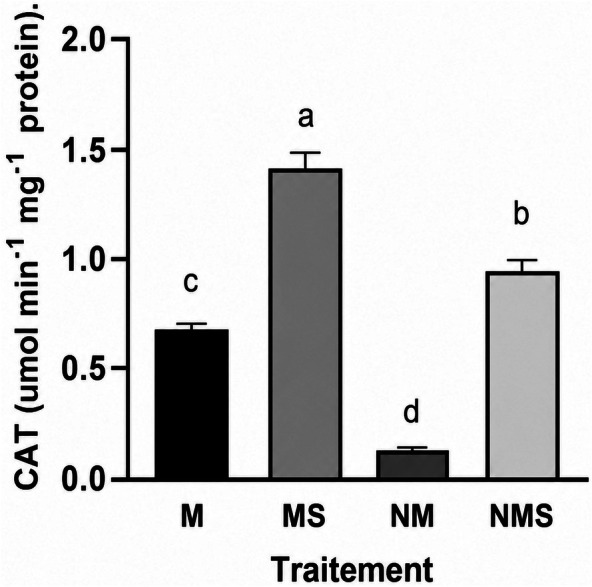
Catalase (CAT) activity in mycorrhizal and non‐mycorrhizal plants under metal stress conditions. M, Mycorrhizal plants; MS, Mycorrhizal and stressed plants; NM, Control plants; NMS, Control and stressed plants. Different letters indicate statistically significant differences (*p* < 0.05).

## 4 Discussion

This study demonstrates that the inoculation of the host plant *L. sativum* with the native AMF from the mining area significantly increases the tolerance level of the host plant to Pb stress. These results indicate that the symbiosis between the host plant and AMF reduces the toxicity of the heavy metal through various physiological and biochemical mechanisms.

This central role of proline as a major osmolyte is emphasized with the observation that proline levels were increased under metal stress and that these levels can be further increased with the inoculation of AMF. This observation underlines the importance of mycorrhizal relationships with the host plant in relation to stress tolerance. It has been reported that the activity of enzymes involved in proline synthesis increases under stress conditions (Abeer et al. 2015). In the current study, mycorrhizal plants (M and MS) showed increased levels of proline compared to non‐mycorrhizal plants. These levels increased with the degree of stress. This increase can be attributed to the osmotic role of proline as well as to the detoxification of reactive oxygen species (ROS). Our results align with Yeganeh et al. (2022), showing that arbuscular mycorrhizal inoculation significantly increases proline accumulation in plants exposed to lead (Pb) stress. Elevated proline levels are associated with improved osmotic adjustment and protection of cellular structures against Pb‐induced oxidative damage. This increase in proline is considered a key adaptive response that helps maintain plant physiological functions under metal stress conditions. Additionally, *Funneliformis mosseae* has been shown to mitigate ROS overproduction by strengthening the host plant's antioxidant defense system, thereby improving overall tolerance and growth in Pb‐contaminated environments. In the current study, the differentiation of mycorrhizal and non‐mycorrhizal plants under stress conditions was evident with statistical differences in proline levels.

Under stress conditions, the increase in soluble sugars in plants constitutes an important adaptive response. Soluble sugars act as osmoprotectants by contributing to the maintenance of cellular water balance, the protection of membranes and proteins, and the limitation of cellular dehydration. Moreover, metal stress induces a high production of reactive oxygen species (ROS), and sugars indirectly participate in the antioxidant defense system by stabilizing cellular structures and reducing oxidative damage. Our study clearly showed a significant increase in soluble sugar content under Pb‐induced metal stress in both mycorrhizal and non‐mycorrhizal plants. This accumulation may be related to osmotic adjustment and protective metabolic responses activated under heavy metal stress. A study conducted by Khan et al. (2021), investigating the effect of metal stress in the absence of mycorrhization, also demonstrated that Pb application increased sugar content in stressed plants. Another study conducted by Yeganeh et al. (2022) on mycorrhizal symbiosis showed that Pb stress increases soluble sugar accumulation in plants, while arbuscular mycorrhizal inoculation further enhances sugar levels, thereby improving osmotic adjustment and tolerance to heavy metal stress, which is consistent with our current study.

The increase in chlorophyll a and b contents observed in mycorrhizal plants subjected to Pb stress could be attributed to improved mineral nutrition and a reduction in metal‐induced oxidative damage. This finding is consistent with the observations of Huang et al. (2018), Bouskout et al. (2022), who reported enhanced photosynthetic pigment contents in mycorrhizal plants exposed to heavy metal stress.

Adverse effects of metallic trace elements on plant growth are well documented, including inhibition of root development, biomass production inhibition, and disruption of chloroplast ultrastructure (Ashraf et al. 2024). Morphological parameters of the plants indicate that Pb stress negatively affects plant growth since there were declines in the fresh weight, dry weight, total plant height, and foliage vigor of the Pb‐stressed plants. However, the performance of the mycorrhizal plants was superior to that of the non‐mycorrhizal plants under both control and Pb‐stressed conditions. These findings support the observations of Ibiang et al. (2018), who reported declines in the dry weight of both mycorrhizal and non‐mycorrhizal plants under zinc stress. However, the treatment effects were not always statistically significant. Better performance of the mycorrhizal plants under Pb stress can be attributed to the various mechanisms by which AMF help the host plant to cope with heavy metal stress. AMF enhance nutrient uptake by the host plant, especially phosphorus (Hnini et al. 2024). Additionally, AMF help to bioaccumulate heavy metals through precipitation, rhizosphere chelation, and sequestration (Hnini et al. 2024).

The enhancement of plant tolerance under metal stress may be attributed to improved antioxidant activity induced by arbuscular mycorrhizal fungi. Previous studies have demonstrated that medicinal plants rich in antioxidant compounds exhibit strong protective activity against oxidative stress (Sarhadi et al. 2026; Shahdadi et al. 2023).

An important mechanism by which AMF mediate metal tolerance is the biosynthesis of glomalin, a glycoprotein that is synthesized by the hyphae of the AMF. Glomalin has a high binding affinity with potentially toxic elements, including lead. This reduces their toxicity in the soil matrix. In the present study, the amounts of easily extracted glomalin were significantly greater in the rhizosphere of mycorrhizal plants compared with those without AMF inoculation, irrespective of the application or otherwise of Pb stress. This study is in agreement with the findings of Meier et al. (2012), who showed that AMF can mitigate the adverse effects of toxic metals on plants by transformation, accumulation, and immobilization of these toxic elements. The authors indicate that hence, *Lactuca sativa* plants inoculated with AMF showed greater Pb tolerance compared with those without AMF inoculation. This is an important mechanism since the accumulation of toxic metals in the soil can significantly affect plant productivity and ultimately influence human health.

Antioxidant defense systems play a vital role in protecting plant cells against oxidative damage caused by stress conditions induced by heavy metal stress. CAT, an antioxidant enzyme, plays a major role in protecting the plant against oxidative damage by decomposing hydrogen peroxide to water and oxygen. In the present study, the activity of CAT was significantly increased in mycorrhizal plants under Pb stress compared to non‐mycorrhizal plants under stress conditions. This shows that mycorrhizal inoculation to the host plant increases the antioxidant defense mechanism to scavenge ROS and protect the plant against stress damage. In non‐mycorrhizal plants under Pb stress conditions, the antioxidant defense mechanism was less effective compared to mycorrhizal plants under stress conditions. In non‐stressed conditions, the activity of CAT was less in all the plants because there was no need to activate the antioxidant defense mechanism. These results are in accordance with the findings of Pooja et al. (2025), which stated that mycorrhization increases the activity of the enzyme catalase and scavenges ROS to stabilize the metabolic process under abiotic stress conditions.

## 5 Conclusion

This study proves the hypothesis that native AMF isolated from the Oued El Heimer mine have the potential to improve the tolerance of *L. sativum* to lead (Pb). Mycorrhizal plants showed improved growth, increased levels of proline and chlorophyll, and higher levels of glomalin and catalase activity than non‐mycorrhizal plants under Pb toxicity. It is evident from the study that mycorrhizal association helps plants cope with metal toxicity through osmoprotectants, metal immobilization, and antioxidant systems. Using native mycorrhizal fungi isolates seems to be a promising approach to the phytoremediation and restoration of metal‐polluted soils.

## Author Contributions

**Fahd A. Nasr:** funding acquisition, resources, visualization. **Ahmed Khalid:** conceptualization, writing – review and editing, supervision, project administration. **Wissame Chafai:** methodology, software. **Mohamed Addi:** conceptualization, writing – review and editing, project administration, supervision, resources, funding acquisition. **Elmostafa Gagou:** methodology, software, formal analysis. **Hanae El Yeznasni:** conceptualization, writing – original draft, investigation, methodology, formal analysis, data curation. **Omar Noman:** writing – review and editing, resources, funding acquisition. **Mashail N. Alzain:** funding acquisition, writing – review and editing, validation.

## Funding

The authors extend their appreciation to Princess Nourah Bint Abdulrahman University Researchers Supporting Project Number PNURSP2026R103, Princess Nourah bint Abdulrahman University, Riyadh, Saudi Arabia.

## Conflicts of Interest

The authors declare no conflicts of interest.

## Data Availability

The data that support the findings of this study are available from the corresponding author upon reasonable request.
